# The Crosstalk between Hypoxia and Innate Immunity in the Development of Obesity-Related Nonalcoholic Fatty Liver Disease

**DOI:** 10.1155/2015/319745

**Published:** 2015-09-28

**Authors:** María Teresa Arias-Loste, Emilio Fábrega, Marcos López-Hoyos, Javier Crespo

**Affiliations:** ^1^Gastroenterology and Hepatology Department, Marqués de Valdecilla University Hospital, 39008 Santander, Spain; ^2^Infection, Immunity and Digestive Pathology Group, Research Institute Marqués de Valdecilla (IDIVAL), 39008 Santander, Spain; ^3^Transplant and Autoimmunity Group, Research Institute Marqués de Valdecilla (IDIVAL), 39008 Santander, Spain; ^4^Immunology Department, Marqués de Valdecilla University Hospital, 39008 Santander, Spain

## Abstract

Nonalcoholic fatty liver disease (NAFLD) has become a major health issue in western countries in parallel with the dramatic increase in the prevalence of obesity and all obesity related conditions, including respiratory diseases as obstructive sleep apnea-hypopnea syndrome (OSAHS). Interestingly, the severity of the liver damage in obesity-related NAFLD has been associated with the concomitant presence of OSAHS. In the presence of obesity, the proinflammatory state in these patients together with intermittent episodes of hypoxia, characteristic of OSAHS pathogenesis, may lead to an enhanced inflammatory response mediated by a positive feedback loop mechanism that implicates HIF-1 and NF*κ*B. Thus, the severity of liver involvement in obese NAFLD patients with a concomitant diagnosis of OSAHS could be explained. In this review, we focus on the molecular mechanisms underlying the hepatic response to chronic intermittent hypoxia and its interaction with innate immunity in obesity-related NAFLD.

## 1. Introduction

Nonalcoholic fatty liver disease (NAFLD) encompasses a wide spectrum of liver damage ranging from simple steatosis to different grades of lobular inflammation, hepatocellular ballooning, and fibrosis (nonalcoholic steatohepatitis; NASH) that may eventually lead to an end stage liver disease [1, 2]. Over the past decade, NAFLD has become a growing medical problem and the main cause of liver disease in industrialized countries [3, 4]. This increase in the prevalence of NAFLD mirrors the emerging epidemic of obesity and metabolic syndrome in this setting [5, 6], which turns into a rise in all obesity-related comorbidities [7], with special interest in different respiratory conditions including the obstructive sleep apnea-hypopnea syndrome (OSAHS) [8]. According to different prevalence studies, in up to 90% of cases of obesity, understood as a body mass index (BMI) higher than 30 kg/m^2^, exists a concomitant NAFLD diagnosis [7, 9, 10].

Recently, the severity of obesity-related NAFLD and more specifically in cases of morbid obesity (BMI higher than 40 kg/m^2^) has been associated with the concomitant diagnosis of OSAHS [11, 12]. But this association is not obesity specific and has been described in population with a BMI lower than 35 kg/m^2^ and in pediatric population, irrespective of the concomitant presence of insulin resistance [13, 14]. The prevalence of OSAHS is higher in men than women and has a direct correlation with the BMI and the hip-waist index [15]. It is estimated that up to 60% of patients with a BMI higher than 30 kg/m^2^ will develop OSAHS [16, 17]. One of the pathophysiological hallmarks of OSAHS is the presence of intermittent episodes of sleeping hypoxia due to the apnea-hypopneas [18]. Intermittent hypoxemia is currently considered a potential major factor contributing to the pathogenesis of OSAHS-related comorbidities. In the long term, these intermittent episodes of hypoxia involve the development of different cellular adaptive mechanisms, in which hepatocytes are included [19–25]. These mechanisms involve the hypoxia-inducible factor (HIF) transcription factors, which are master regulators of the cellular response to the hypoxia and coordinate a transcriptional program that guarantee an adequate metabolic, vascular, and functional response to oxygen deficiency [26]. Moreover, recently how HIF transcription factors can be key elements also in the control of immune cell metabolism and functionality has been described [27].

Therefore, the aim of this review is to go through the molecular mechanism underlying the hepatic response to chronic intermittent hypoxia and to discuss its interaction with innate immunity in the setting of obesity-related NAFLD.

## 2. Cellular Consequences of Hypoxia

### 2.1. Hypoxia-Inducible Factor

The cellular adaptation to hypoxia lies on the HIF transcription factor that is made up of the HIF-1*β* subunit, which is constitutively expressed, and two *α* subunits: HIF-1*α* and HIF-2*α* [28]. This transcription factor regulates the expression of multiple genes involved in oxygen transportation, angiogenesis, proliferation, and metabolism, enabling a cell to counteract a hypoxic environment [26].

Under normoxic conditions, the iron-dependent enzymes prolyl hydroxylases (PHDs) present in the cytoplasm are active and hydroxylate HIF-*α*, leading to its proteosomic degradation mediated by von Hippel-Lindau (VHL) dependent ubiquitination. Contrary to this mechanism, when oxygen levels decrease, PHDs are inactive and therefore HIF-*α* can accumulate, stabilize, and, eventually, translocate to the nucleus. Another level of regulation is constituted by the factor inhibiting HIF (FIH). FIH hydroxylates asparaginyl residues in HIF-1*α* and HIF-2*α*, reaching the blockade of protein interactions between HIF-*α* and different coactivators like P300 that form the transcriptional complex. Oxygen acts as a cofactor of FIH and therefore when oxygen is scarce, the ability of FIH to hydroxylate HIF residues decreases leading again to HIF-*α* accumulation, stabilization, and nuclear translocation [29, 30].

### 2.2. HIF and Innate Immunity: Association with Toll-Like Receptors

Besides this posttranscriptional and oxygen-dependent HIF regulation, there are other mechanisms of HIF regulation at a transcriptional level that are oxygen-independent and work under inflammatory, infectious, or oxidative stress conditions [31]. In this sense, how bacterial lipopolysaccharide (LPS), a cell membrane component of Gram-negative bacteria, can increase HIF-1*α* transcription has been previously shown [32]. Bacterial products are recognized by Toll-like receptors (TLRs) expressed on myeloid cells. The downstream signaling of TLR involves NF*κ*B, which plays a central role in regulating the immune response to infection and inflammation, and also induces a HIF-1*α* mRNA transcriptional response. Moreover, key inflammatory cytokines, as tumor necrosis factor alpha (TNF-*α*), can induce HIF-1*α* expression in innate immune cells [33]. Under oxygen shortage conditions, the expression and signaling transduction of TLRs increase, resulting in an amplification of the NF*κ*B pathway [34]. Thus, the innate immune response is enhanced and amplified.

## 3. The Role of Hypoxia in Obesity-Related Nonalcoholic Fatty Liver Disease

### 3.1. Effect of Hypoxia in the Liver

The repercussion of intermittent hypoxia in the liver has been assessed in different animal models addressing its consequences mainly in terms of hepatocyte injury [22, 24], lipid accumulation [21], and endothelial dysfunction [20]. First, Savransky et al. demonstrated in animal models that intermittent hypoxia is able to induce mild liver injury, being its main effect to predispose the liver to the hepatocellular damage seen in different settings, as alcohol intake, drug-induced hepatotoxicity, or the low-grade inflammation present in the metabolic syndrome [25].

Regarding NAFLD and NASH, how literature supports a key role of hypoxia in lipid metabolism is of interest [35]. Importantly, Piguet and collaborators showed in mice models of nonalcoholic steatohepatitis how hypoxia upregulates genes involved in lipogenesis, like SREBP-1c (sterol-regulatory-element-binding protein-1c), PPAR-gamma (peroxisome-proliferator-activated receptor-gamma), ACC1 (acetyl-CoA carboxylase 1), or ACC2 (acetyl-CoA carboxylase 2), whereas genes involved in lipid metabolism as PPAR-alpha (peroxisome-proliferator-activated receptor-alpha) and CPT-1 (carnitine palmitoyltransferase-1) were downregulated [23]. Moreover, hypoxia has been also associated with upregulation of genes involved in lipid uptake and lipid droplet formation [36, 37]. Several works have focused on the role of HIF signal-transduction pathway in lipid metabolism and liver damage under hypoxic conditions. Thus, Rankin et al. showed in different animal models that HIF-2*α* acts as a key regulator of hepatic lipid metabolism, as it impairs fatty acid *β*-oxidation, increases lipid storage capacity, and decreases lipogenic gene expression, all of these resulting in the development of severe hepatic steatosis [38]. Later, Qu and collaborators working with animal models that overexpressed HIF through VHL-disruption demonstrated that, besides a time-dependent effect of HIF on lipogenic gene expression, a rapid increase in proinflammatory cytokines and fibrogenic gene expression was also observed in hypoxia [39].

Nevertheless, conflicting results have been observed regarding HIF-1*α* downstream signaling in the liver. More than a decade ago, Yun et al. [40] elegantly showed in an animal model how hypoxia inhibits adipogenesis via HIF-1 through repression of* PPARγ2* promoter activation by the HIF-1-regulated gene* DEC1/Stra13*. More recently, Nishiyama et al. have suggested that HIF-1*α* may act as a protective factor against lipid accumulation in ethanol-induced liver damage through activation of* DEC1* [41]. These results are in agreement with different studies that have shown beneficial effects of short-term intermittent hypoxia in terms of endothelial function, mitochondrial activity, and steatosis development [42, 43]. In contrast, Nath and collaborators [22] showed that alcohol intake leads to hepatic fat accumulation through HIF-1*α* activation in mice engineered with hepatocyte-specific HIF-1*α* activation, whereas hepatocyte-specific deletion of HIF-1*α* conferred protection from alcohol. Moreover, how the coexistence of alcohol and lipopolysaccharide (LPS) mediated liver damage enhanced hepatic steatosis through induction of monocyte chemoattractant protein 1 (MCP1) via HIF-1*α* activation was demonstrated.

The effect that hypoxia exerts on insulin signaling is also of great interest, as insulin resistance is a characteristic hallmark in fatty liver development. In this sense, previous studies in liver specific* Phd3* (an isoform of prolyl hydroxylases) knockout mice have suggested that stabilization of hepatic HIF-2*α* turns out into improved insulin sensitivity [44]. In line with this study, Wei et al. [45] demonstrated a link between HIF-2*α* expression, but not HIF-1*α* in murine liver, and an increase in hepatic insulin sensitivity through the induction of insulin receptor substrate 2. This study also pointed out the distinct roles in hepatic metabolism for HIF-1*α*, which promotes glycolysis, versus HIF-2*α*, which suppresses gluconeogenesis.

Finally, hypoxia is a major feature in many solid tumors, including hepatocellular carcinoma (HCC). It can promote tumor progression in a mechanism at least partially promoted by HIF-1 that activates hypoxia-responsive genes that will interplay in the natural history of HCC. These genes will mediate in multiple aspects of proliferation, metabolism, angiogenesis, invasion, metastasis, or therapy resistance [46–48].

### 3.2. Consequences of Hypoxia in Adipose Tissue

Expanded subcutaneous and visceral fat is a hallmark of obesity. As a result of this, the enlarged adipose tissue produces and releases different proinflammatory cytokines and adipokines that will be, at least in part, responsible for the low-grade proinflammatory state associated with obesity [49–51]. Recently, it has been suggested that fat inflammation can also be triggered by hypoxic conditions [52]. The reduction in oxygen availability in adipose tissue of obese patients, irrespective of concomitant respiratory conditions, responds to different factors. When adipocytes get bigger, the oxygen diffusion can be impaired, oxygen supply may be reduced due to a decrease in capillary density, and finally and as shown in a recent study obesity and high-fat diet can also increase oxygen consumption in adipocytes, probably due to uncoupled respiration induced by free-fatty acids [53–56]. Moreover, oxygen shortage in adipose tissue will lead not only to the production of adipokines and proinflammatory cytokines [57], but also to an impaired glucose homeostasis and lipid metabolism [58, 59]. Furthermore, hypoxia also inhibits adipogenic differentiation, which favors adipocyte enlargement, with the perpetuation of the situation [40]. Taking together all this data, it can be assumed that adipose tissue hypoxia is a major driver of cardiovascular and metabolic entities associated with obesity and mediated by inflammation. According to this, how the deletion of HIF-1*α* in adipocytes enhances glucagon-like peptide-1 secretion and reduces adipose tissue inflammation has been shown, improving glucose tolerance. This points out a potential new target in obesity-related comorbidities [60].

## 4. Innate Immunity in Nonalcoholic Fatty Liver Disease: Role of Toll-Like Receptors

The high prevalence of cardiovascular comorbidity in NASH patients is assumed to be associated with a low-grade proinflammatory state [61], initiated mainly in the expanded visceral fat and at least partially perpetuated in the liver [62, 63]. Activation of inflammatory pathways in both fat tissue and liver includes those dependent on TLRs and merges mainly in the activation of NF*κ*B [64–66]. TLRs are pattern recognition receptors that characteristically perceive pathogenic microorganisms and bacterial-derived molecules, leading to the production of different proinflammatory cytokines [67, 68]. Among the thirteen different TLRs that have been described in mammals, only TLR2, TLR4, TLR5, and TLR9 have been documented to clearly associate with NAFLD pathogenesis and progression [69–74].

The role of TLR4 has been extensively assessed in both animal models and humans. TLR4 forms a complex with MD2 on the cell surface that specifically binds and responds to bacterial LPS [75, 76]. Circulating LPS levels appear increased in animal models and patients with a diagnosis of NAFLD, but also in patients with insulin resistance [77–79]. This endotoxemia may be explained by different factors associated with gut microbiota, gut permeability, and high-fat diet [80–84]. Furthermore, LPS can also be increased in patients undergoing intestinal bypass or in patients with total parenteral nutrition, resulting in the development of steatosis that can occur irrespective of the presence of other features of the metabolic syndrome [85–87]. The determinant role of this TLR4-LPS interaction and downstream signalling in NAFLD pathogenesis has been documented in TLR4 mutant mice in which, besides the presence of similar plasmatic levels of LPS compared to wild type animals, the expression of proinflammatory cytokines was suppressed and neither NAFLD nor insulin resistance was developed [70, 88].

Additionally, it is important to note that TLR4 can also respond to free-fatty acids (FFA). In this line, a recent study in human monocytes has demonstrated that FFA can activate TLR4 in the presence of high levels of glucose [89]. This phenomenon can be explained due to the fact that lauric acid, a medium chain fatty acid also present in LPS, can exert downstream signaling dependent on TLR4 in macrophages [90, 91]. Thus, saturated fatty acids, whose levels are frequently increased in plasma of obese patients, can play a key role in the development of diet-induced IR, as has been previously reported [92].

Different studies have implicated TLR4 in the pathogenesis of HCC in NAFLD [93, 94]. In the last decade, a number of studies have addressed the implication of proinflammatory signaling transduction and carcinogenesis [95–97]. In this line, Dapito et al. established in a recent study the importance of the LPS-TLR4 pathway in hepatocarcinogenesis in several genetically different mouse lines in which HCC was induced following different protocols. This way, they could demonstrate that inactivating TLR4 had no effect on HCC incidence but significantly reduced tumor size and number. Additionally, in wild type mice intoxicated with DEN/CCl4, they found that continuous administration of LPS increased tumor number and size [93]. Although in this study no association between TLR4 pathway and incidence was observed, previously Yu et al. did find this association [98]. This discrepancy might be explained by the different source of the LPS used as TLR4-agonist implicated in HCC development.

Regarding other TLRs implicated in NAFLD pathogenesis, TLR2 is also a cell surface receptor, which is involved in the recognition of a wide range of pathogen-associated molecular patterns (PAMPs) including peptidoglycan, a component of the cell surface of Gram-positive bacteria [75]. These subtypes of bacteria include Firmicutes, whose increase has been reported in animal models and humans subjected to a high-fat diet [99]. Furthermore, this dysbiosis has also been associated with NAFLD [100]. The connection between gut microbiota and NAFLD has been previously addressed in different studies. Thus, research on mice on a high-fat diet has shown that the blockade of TLR2 signaling prevents the development of insulin resistance [80]. Moreover, TLR2 deficient mice with a dietary-induced NASH do not develop steatohepatitis and display lower expression of proinflammatory cytokines [101, 102].

Briefly, according to TLR5 and TLR9, whereas TLR5 is another cell membrane receptor, TLR9 is the only intracellular TLR implicated in NAFLD pathogenesis. TLR5 recognizes the flagellin protein component of bacterial flagella and has not been directly associated with NASH but with dysbiosis and related metabolic syndrome [74, 103, 104]. Thus its role in NAFLD pathogenesis remains to be clarified. TLR9 recognizes unmethylated DNA motifs that are frequently present in bacteria and viruses but rare in mammalian cells. Studies in TLR9 (−/−) mouse models of NASH have shown that TLR9 downstream signaling is associated with NASH severity and fibrosis by the production of IL-1*β* [105]. Finally, in animal models of colitis with high portal levels of LPS, an increase in hepatic TLR9 mRNA levels associated with hepatic steatosis, inflammation, and fibrosis has been documented [106].

## 5. Interaction between Hypoxia and Inflammatory Pathways in the Development of Obesity-Related Nonalcoholic Fatty Liver Disease

Lipid accumulation in the form mainly of triglycerides is the distinctive trait of NAFLD. In less than 25% of cases, this deposit leads to a variable degree of lobular inflammation and hepatocellular injury and, consequently, a higher risk of disease progression [1, 107]. The reasons why liver diseases eventually progress to more severe forms only in some patients remain to be fully elucidated and are subject of study and debate. Thus, in 1998 Day and James expound the classic “two-hit” hypothesis of NAFLD pathogenesis [108]. According to this hypothesis, the disease pathogenesis is sequential, with a first hit consisting in an excessive intrahepatic lipid accumulation, which can be followed by a second hit, resulting in inflammation, hepatocellular damage, and, therefore, NASH. A decade later, Tilg and Moschen proposed the so-called “multiple parallel hits” hypothesis [109] where it was suggested that disease pathogenesis may not be sequential. According to this work, inflammation could precede steatosis in certain scenarios and NASH development could be the consequence of different parallel hits derived from the gut and/or the adipose tissue. Interestingly, in this setting, many cytokines, adipokines, and inflammatory signaling networks mainly regulated by innate immunity emerged as key elements in the disease progression.

As previously mentioned, the activation of inflammatory pathways in NAFLD, not only in the liver, but also in the adipose tissue, with eventual reflection in the liver, includes those dependent on TLRs and merges mainly in the activation of NF*κ*B [64–66]. Importantly, it has been demonstrated that NF*κ*B is a critical transcriptional activator of HIF-1*α* and basal NF*κ*B activity is required for HIF-1*α* protein accumulation under hypoxic conditions [110]. Moreover, hypoxia may modulate innate immune response in the setting of an infection or inflammation by transcriptional regulation of TLRs expression and function via HIF-1*α* [34, 111]. Thus, the inflammatory scenario present in NASH may boost and possibly perpetuates the consequences of hypoxia in both the liver and adipose tissue. This overexpression of HIF-1*α* under inflammatory conditions can possibly explain the divergences seen in previously mentioned studies assessing its role in NAFLD, since the consequences of hypoxia may differ in the setting of simple steatosis compared to NASH [22, 41]. Furthermore, proinflammatory cytokines as IL-6, IL-1, and TNF-*α* will also contribute to the “vicious circle” of steatosis and inflammation by increasing the lipid deposits through a mechanism that implicates TLRs and leads to insulin resistance [49, 105, 112, 113].

Summarizing, as previously suggested by Savransky et al. [25], chronic intermittent hypoxia can be considered to predispose to liver injury sensitizing the liver to a second insult. In the presence of obesity, the proinflammatory state in these patients together with intermittent episodes of hypoxia may lead to an enhanced inflammatory response mediated by a positive feedback loop mechanism that implicates HIF-1 and NF*κ*B, which could explain the presence of more severe forms of liver involvement in obesity-related NAFLD in the presence of OSAHS [11]. Thus, hypoxia could be considered as another “hit” among the “multiple parallel hits” that have been suggested as responsible for disease pathogenesis.

## 6. Concluding Remarks

Obesity and obesity-related comorbidities, including respiratory conditions as OSAHS, are dramatically increasing in the last decades. In this scenario, different pathogenic mechanisms coexist with a complex molecular signaling network in which inflammation plays a preeminent role. In line with this, it is important to better define and understand the interaction of different etiologies in the same individual that eventually may lead to insulin resistance, metabolic syndrome, and liver injury. In this review, we have focused on the molecular mechanism underlying inflammatory pathways in insulin resistance and NAFLD triggered by hypoxic conditions, so frequent in obesity. These two elements are modulated by multiple factors like diet, microbiota, or genetic background that can intensify and even perpetuate the inflammatory response. Importantly, inflammation mediated by innate immunity and hypoxia may lead to the development of HCC, among other tumors. From a further and deeper understanding of the molecular basis underlaying HCC pathogenesis new approaches and molecular targets will be developed.
